# PD33 Exploring The Influence Of Patient Preference Information In Health Technology Assessment: Insights From Mock Deliberations With Agencies

**DOI:** 10.1017/S0266462325101670

**Published:** 2025-12-29

**Authors:** Carina Oedingen, Barry Liden, Simon Fifer, Deborah A. Marshall, Maya Joshi, Catherine Koola Fischer, Mandy Tonkinson, Alissa Hanna, Mickael Hiligsmann, Evi Germeni, Mo Zhou, Barry Stein, Harry Kotlarz

## Abstract

**Introduction:**

Internationally, there is a growing drive to integrate patient preference information (PPI) into health technology assessment (HTA) decision-making. However, it is unclear how PPI is used in real-world contexts and the extent to which it can reshape and influence HTA decisions. Understanding how PPI can be meaningfully used in HTA is fundamental for supporting its practical application and increasing trust in its use.

**Methods:**

The aim was to enhance understanding of how HTA committees interpret and incorporate PPI into decision-making. Mock deliberation workshops were conducted by members of the HTAi Patient Preference Project Subcommittee with experts from four HTA agencies. For the workshops, a mock evidence package was developed that included PPI evidence alongside key clinical and economic evidence to simulate decision-making processes. The mock deliberations were analyzed using thematic analysis.

**Results:**

The mock deliberation workshops (25 participants) highlighted both opportunities and challenges in integrating PPI into HTA processes. While PPI was acknowledged as valuable evidence, its influence on decision-making in the scenarios varied from being cited by a few participants as informing the decision to most participants indicating the evidence was not persuasive. Participants pointed out the need for more structured guidance on how to incorporate PPI alongside clinical and economic evidence. The findings highlighted the importance of standardizing the path to integration, as well as the content and format of PPI, to support consistent interpretation and application in HTA.

**Conclusions:**

By examining how PPI is weighed alongside clinical and economic evidence, the project offered valuable insights for stakeholders aiming to generate more pertinent PPI studies for incorporation into the decision-making process. The key takeaways for HTA agencies included strategies for effectively balancing PPI with clinical and economic evidence, highlighting a shift toward incorporating more diverse and inclusive forms of evidence.

